# Inequality and Influencing Factors of Spatial Accessibility of Medical Facilities in Rural Areas of China: A Case Study of Henan Province

**DOI:** 10.3390/ijerph16101833

**Published:** 2019-05-23

**Authors:** Shirui Liu, Yaochen Qin, Yanan Xu

**Affiliations:** 1 College of Environment and Planning, Henan University, Kaifeng 475004, China; liushirui@henu.edu.cn; 2 Key Laboratory of Geospatial Technology for the Middle and Low Yellow River Regions, Henan University, Kaifeng 475004, China; 3 College of Resources and Environment, Henan University of Economics and Law, Zhengzhou 450046, China; ynxu137@163.com

**Keywords:** spatial accessibility, rural area, spatial pattern, GWR, Henan Province

## Abstract

The equalization of medical services has received increasing attention, and improving the accessibility of medical facilities in rural areas is key for the realization of fairness with regard to medical services. This study studies the rural areas of Henan Province, China, and uses unincorporated villages as the basic unit. The spatial pattern of accessibility in rural areas was comprehensively analyzed via geographic information system spatial analysis and coefficient of variation. The spatial heterogeneity of relevant influencing factors was assessed by using the geographically weighted regression model. The results show that: (1) The distance cost of medical treatment in rural areas is normally distributed, and most areas are within a range of 2–6 km. (2) The accessibility in rural areas has clear spatial differences, is significantly affected by terrain, and shows characteristics of significant spatial agglomeration. The eastern and central regions have good spatial accessibility, while the western regions have poor spatial accessibility. Furthermore, regions with poor accessibility are mainly located in mountainous areas. (3) The spatial equilibrium of accessibility follows a pattern of gradual deterioration from east to west. The better accessibility-unbalanced type is mostly located in the center of Henan Province, while the poor accessibility-unbalanced type is concentrated in mountainous areas. (4) The area, elevation, residential density, and per capita industrial output are positively correlated with spatial accessibility, while road network density and population density are negatively correlated.

## 1. Introduction

With economic development and the improvement of the quality of life of residents, their demand for health and hygiene is continually increasing. For a long time, the structure of medical and health resources in both urban and rural areas of China has been unreasonable [1]. The geographical spatial layout of medical and health institutions suffers from a lack of effective overall planning, and the spatial distribution of medical facilities is unbalanced. This results in an increasing inconsistency between the multi-level medical and health demand and the apparent shortage of medical facilities [2]. Due to factors of system, urbanization, and economy, medical resources are mainly concentrated in urban areas, while the public health investment in rural areas is clearly inadequate [1,3]. The inconvenience connected with seeking medical treatment has restricted the improvement of resident health in rural areas of China for a long time [4]. Therefore, increasing attention focuses on the equalization of medical services.

Accessibility is a decisive factor that affects the level of medical service equalization. Accessibility of medical facilities not only reflects the opportunity and convenience of the public to access medical services, but also affects the improvement of the quality of life of residents [5]. The spatial allocation and accessibility of medical facilities is theoretically and practically significant to gain an understanding of the current situation of medical resources. It will also aid the identification of areas that suffer from a lack of medical infrastructure, and the optimization of the spatial layout of medical facilities [6].

Accessibility is affected by both spatial factors and non-spatial factors: spatial factors mainly include the distance from the residential area to the hospital, which determine the distance or time cost of the accessibility of medical care for residents. Non-spatial factors include the attribute characteristics of medical facilities (e.g., scale, grade, and quantity), as well as the self-attributes of residents in need of medical treatment (economic income, medical preference, and number of residents) [7]. In the current diagnosis and treatment system of China, medical accessibility is predominantly affected by spatial factors [8].

The methods to measure spatial accessibility mainly include the nearest distance method, the Huff model, the kernel density method, the potential model, and the two-step floating catchment area (2SFCA) method. Of these methods, both the potential model and the 2SFCA method have been widely used and developed so far, since they consider scale factors of both the supply-side and the demand-side, as well as the distance cost factor. In 1959, Hansen proposed the gravity model as a measure of accessibility that considers both the supply-side scale and distance cost. Joseph and Bantock improved the gravity model, added the population scale factor, and introduced the concept of the gravity potential model [9]. Subsequently, scholars applied the gravity potential model to geography and developed it into a potential model. Based on this, Song et al. added the influence coefficient of the scale of medical facilities to indicate the influence medical facilities exert on the behavior of residents that seek medical treatment in residential areas [10]. The 2SFCA was first proposed by Radke, further improved by Luo and Wang and was named 2SFCA [11]. The 2SFCA uses dichotomy to integrate distance attenuation, i.e., the accessibility is identical within a search radius threshold range, but completely unreachable outside this search radius range. Currently, four types of extended forms of the 2SFCA method have been developed: an extension of the distance attenuation function [12,13,14,15], an extension of the search radius [16,17,18], an extension of the demand or supply competition [19,20,21,22], and an extension based on the travel mode [23,24]. Both the potential model and the 2SFCA method have limitations, and use demographic units as analytical units. Currently, the demographics of China are all above the scale of townships and streets. Therefore, the potential model and the 2SFCA method are not suitable for refined research [25].

Based on the measurement method of accessibility, scholars have conducted considerable applied research with the following main aims: (1) Determine accessibility of medical treatment for different groups. Through a survey of 42 states in the United States, Donald and Hoenig found a lack of walking access to medical facilities around residential areas for older people with disabilities; therefore, they chose medical care at their homes [26]. Wu et al. suggested that a community care policy assessment requires a comprehensive and weighted calculation process, including the elderly walkability distance-decay factor, demand population, and supplier loading [27]. Tao et al. reported that the accessibility of medical facilities of the registered population was better than that of the floating population [28]. (2) Examine accessibility of medical treatment under different travel modes. Meng et al. compared the accessibility of medical services between the two travel modes of ground transportation and rail transit. The results showed that the time cost for medical treatment of the rail transit mode was significantly higher than that of the ground transportation mode [29]. Zhong et al. studied the spatial accessibility of medical facilities in response to different referral rates based on the 2SFCA method. The reported results showed that for a 60% referral rate, the spatial accessibility of medical facilities considering underground traffic was about 9.81% higher than without considering underground traffic [30]. (3) Study differences in accessibility of hospitals at different scales. Huang et al. studied the medical service function of three types of hospitals, and ordered their accessibility from strong to weak as clinic, general hospital, and specialized hospital [31]. Based on the improved potential model, Cheng et al. reported that the spatial accessibility of tertiary hospitals was better than that of second and first level hospitals [32]. Currently, scholars mainly focus on medical accessibility in urban areas. Most of the research areas are within a single city, thus ignoring residents who choose to receive medical treatment across administrative areas. However, the optimal allocation of local areas is typically affected by macro policies in larger regions [33]. Due to the long-term dual urban-rural structure in China, the per capita income difference between urban and rural residents is considerable. Furthermore, high-quality medical resources are typically concentrated in urban areas, resulting in problems explained with "difficulty to see a doctor" and "expensive to see a doctor" in rural areas. Improving the accessibility of medical facilities for residents of rural areas has become one of the key issues that need to be overcome to realize fairness in the provision of medical services [34].

To this regard, this study investigated Henan Province, which is the province with the largest rural population in China. Based on geographic information system (GIS) network analysis and the spatial analysis method, the spatial differentiation characteristics of accessibility and their equilibrium in rural areas were analyzed. The specific research objectives were to: (1) Explore the spatial difference of accessibility of medical facilities in rural areas of Henan Province, and to analyze the equilibrium degree within and between townships. (2) Employ spatial statistical analysis methods to quantify the spatial pattern of medical accessibility, and identify the spatial agglomeration areas and a number of special areas. (3) Explore the influencing factors of accessibility of medical facilities in rural areas from the three aspects of nature, society, and economy, and to reveals their spatial heterogeneity. To explore the clustering patterns of spatial accessibility in the geographical space, spatial autocorrelation was employed to assess more specific information related to the spatial imbalance of hospital accessibility. Spatial autocorrelation includes global autocorrelation and local autocorrelation. Therefore, this study analyzed the clustering patterns of spatial accessibility from these two aspects, and identify the spatial agglomeration areas and a number of special areas. The spatial heterogeneity of influencing factors was revealed via by GWR model. This study provides a reference for the optimization of regional medical resource allocation and for the layout planning of public medical facilities.

## 2. Materials and Methods

### 2.1. Study Area and Data Sources

**Figure 1:**
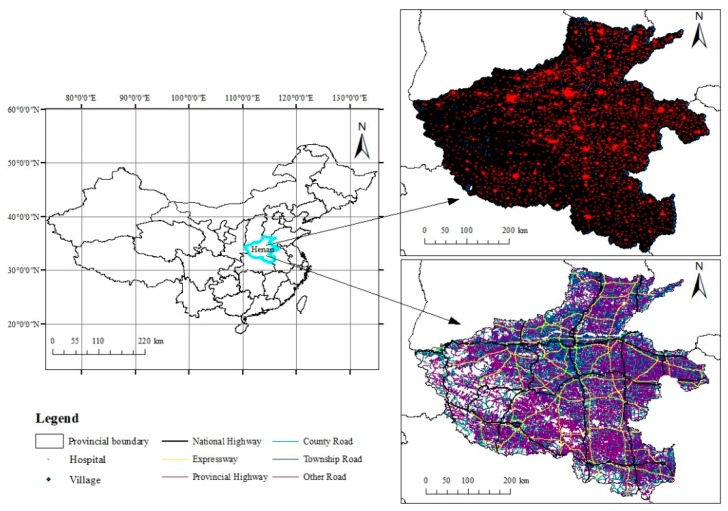
Road network and spatial distribution of villages and hospitals in the study area (Henan Province).

This paper uses the unincorporated village as the basic research unit. The unincorporated village also called a natural village, and has naturally formed by villagers living together in a natural environment for a long time. These are naturally settled by families, households, clans, or other reasons. The unincorporated village is the most basic unit of rural settlement, and it is also a relatively independent residential area. Spatial location data of unincorporated villages were obtained from the points of interest (POI) of Baidu Maps [35]. Baidu Maps is a desktop and mobile web mapping service application and technology provided by the Baidu company. It is similar to Google Maps, and can obtain data such as traffic routes and spatial locations of public facilities. A total of 203801 villages with spatial location data (including 38179 villages and neighborhood committees in urban areas) in Henan Province were obtained. Similarly, through POI of Baidu Maps, the location data of 5321 hospitals were obtained. These did not include village clinics, specialized hospitals, or rehabilitation hospitals (Figure 1).

The administrative boundary vector data and 1:30 m digital elevation model (DEM) data were obtained from the Data Center of the Lower Yellow River Regions [36]. The road network data was obtained from the open street map [37], as shown in Figure 1. The statistical data, including population, economic, and other indicators were obtained from the China Statistical Yearbook (Township Volume) [38]. All data were updated to the year 2018. Rural areas refer to places where workers are mainly engaged in agricultural production and live together. Rural areas are also known as non-urbanized areas. Compared to cities with their highly concentrated population, the population of rural areas is scattered. Therefore, rural areas in this study refer to areas outside municipal districts and county towns. 1609 townships in Henan Province belong to rural areas.

### 2.2. Methods

#### 2.2.1. Spatial Accessibility Calculation

This study used the nearest distance method to measure the spatial accessibility in rural areas. The nearest distance method measures the shortest path from a settlement to a hospital. This shortest path contains both the fastest path in time and the lowest cost of transportation. The nearest service impedance captures the proximity between the population and the location of the service. The proximity of services relative to the location of the population is one of the important characteristics of accessibility [33]. Studying accessibility by the nearest distance method can directly reflect the spatial fairness and convenience of residents’ access to medical services in rural areas [39]. The nearest distance method can identify the distance cost of the residents to the hospital: a higher distance cost indicates lower accessibility. The overall accessibility of the township is expressed by the mean accessibility of all villages in administrative divisions, as shown in Equation (1):

(1) $$T_{j}=\frac{\sum_{i=1}^{n}F_{i}}{n}$$

where *n* represents the number of villages in the area *j*, Fi represents the distance cost from the settlement *i* to the nearest hospital. The higher the *T_j_* value, the worse the accessibility of region *j*. In addition, to compare the difference of spatial accessibilities between rural and urban areas, the distance cost between residential areas and hospitals in urban areas was calculated with the same method. The overall medical accessibility in urban areas was obtained by using the mean value method. The nearest distance method was implemented in ArcGIS 10.2 software. By creating OD matrix, the shortest path from village to hospital can be obtained, i.e., the distance cost from village to hospital, and then the average distance cost at the township scale can be obtained.

#### 2.2.2. Coefficient of Variation

In order to explore the spatial inequality of medical accessibility in rural areas, coefficient of variation (CV) method was used to evaluate it. The CV is a statistic that measures the degree of dispersion between different observations in a data set. In this study, CV was used to evaluate the equilibrium degree of each township. The formula is as follows:

(2) $$CV_{i}=\frac{\sigma_{i}}{\mu_{i}}$$

where *σ_i_* represents the standard deviation of accessibility of all settlements in area *i*, and *μ_i_* represents the average value of accessibility of all settlements in area *i*.

To further explore the spatial distribution characteristics of accessibility and its equilibrium in rural areas, both were divided as follows: When the distance cost of medical treatment is less than or equal to 5000 m, the accessibility is defined as good, but when the distance cost of medical treatment exceeds 5000 m, the accessibility is defined as poor. A CV of less than or equal to 0.5 indicates a relatively balanced situation, while a CV exceeding 0.5 indicates imbalance. Therefore, the accessibility-equilibrium in rural areas of Henan Province can be divided into four types of categories.

#### 2.2.3. Spatial Autocorrelation

To explore the clustering patterns of spatial accessibility in the geographical space, spatial autocorrelation was employed to assess more specific information related to the spatial imbalance of hospital accessibility, and identify the spatial agglomeration areas and a number of special areas. The Moran’s I index is typically used to judge whether a spatial correlation exists between an element in space and an element in its adjacent space. Here, the Global Moran’s I index represents the global spatial autocorrelation of the object of study, which is calculated as follows:

(3) $$I=\frac{n}{S_{0}}\frac{\sum_{i=1}^{n}\sum_{j=1}^{n}W_{ij}Z_{i}Z_{j}}{\sum_{i=1}^{n}Z_{i}{}^{2}}$$

(4) $$S_{0}=\overset{n}{\underset{i=1}{\sum }}\overset{n}{\underset{j=1}{\sum }}W_{ij}Z_{i}Z_{j}$$

where *n* represents the total number of study areas, *S_0_* represents the aggregation of all spatial weights, *Z_i_* represents the deviation between the attribute value of the region i and the average value, and *W_ij_* represents the spatial weight between region *i* and *j*. If *i* and *j* are adjacent, *W_ij_* = 1, otherwise *W_ij_* = 0. The range of the Moran’s I index is approximately scored from −1 to +1. If the value of *I* exceeds 0, this indicates that the study object is spatially positively autocorrelated. A larger value indicates a stronger spatial aggregation. If the value of *I* is less than 0, the subjects show a spatially negative autocorrelation, and a smaller value indicates a stronger spatial aggregation. *I* = 0 indicates spatially uncorrelated data.

In addition to the analysis of global characteristics, several local features of spatial accessibility also need to be investigated. The Local Moran’s I index represents the local spatial autocorrelation of an attribute of a spatial object. It decomposes the Global Moran’s I method into the local space, i.e., for each object in space:

(5) $$I_{i}=\sum W_{ij}^{\prime }Z_{i}Z_{j}$$

The significance of spatial autocorrelation was judged by the standardized statistic *Z*. At a confidence level of 0.05, the absolute value of *Z* equals 1.96. When the absolute value of *Z* exceeds 1.96, the spatial autocorrelation is significant.

#### 2.2.4. Geographically Weighted Regression

To explore the influencing factors and spatial heterogeneity of accessibility of medical facilities, the geographical weighted regression (GWR) model was utilized. The GWR embeds the geographical location of the data into parameters based on a traditional regression, so that the parameters can be estimated locally. By establishing the local regression equation at each point in the spatial position, the spatial variation and related driving factors of the research object can be explored at a specific scale. The GWR model can optimize the weight in the local area, so that the influence of independent variables on dependent variables corresponds to a regression coefficient value at each sample point. This reflects the spatial heterogeneity between dependent variables and independent variables, and truly depicts the specific situation of the influence of independent variables on dependent variables. Therefore, the GWR has an advantage for the study of the local effect of the spatial object, and its model is as follows:

(6) $$y_{i}=\beta_{0}\left(u_{i},v_{i}\right)+\overset{k}{\underset{i=1}{\sum }}\beta_{k}\left(u_{i},v_{i}\right)x_{ik}+\varepsilon_{i}$$

where *y_i_* represents the dependent variable of the sample point *i*, *x_ik_* represents the observation of the k-th variable on the sample point *i*, *(u_i_, v_i_)* represents the geospatial coordinate of the sample point i, *β_0_ (u_i_, v_i_)* represents a constant regression term, *β_k_ (ui, vi) x_ik_* represents the k-th regression parameter on the sample point *I*, and *ε_i_* represents the error term.

Before using the GWR model, it is necessary to conduct an ordinary least square (OLS) regression to the variable. The results of this OLS regression are then used to determine whether multiple collinearity exists between independent variables. In addition, the AICc values of the output results of both models need to be compared. According to Fotheringham et al., if the AICc value of the GWR model is 3 or less than that of the OLS model, the GWR model is suitable [40]. This study constructed the GWR model by using ArcGIS 10.2 software. In this study, the adaptive Gaussian kernel type was selected, the optimal bandwidth was determined via the AICc minimization criterion, and the spatial weight matrix was constructed via the edge adjacency method.

## 3. Results

### 3.1. Analysis of Spatial Accessibility in Rural Areas

**Figure 2:**
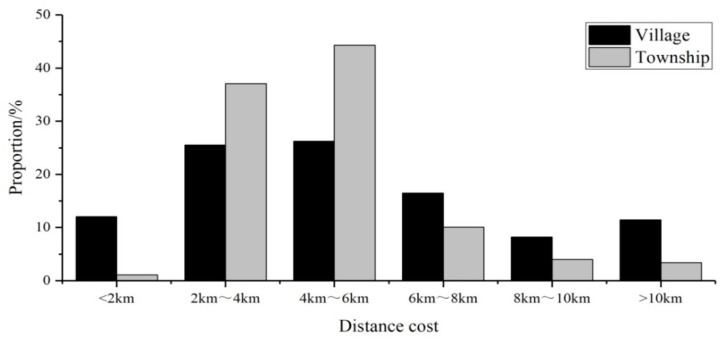
Distribution of distance cost of medical treatment in rural areas of Henan Province.

**Figure 3:**
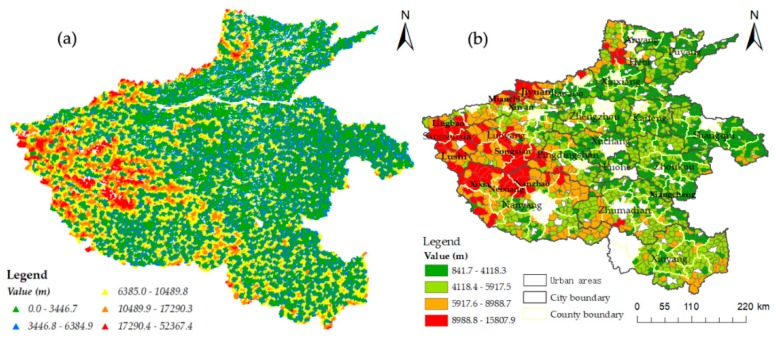
Medical accessibility in rural areas of Henan Province: (**a**) distance cost of medical treatment in villages; (**b**) spatial distribution of medical accessibility in townships.

Based on the nearest distance method, the distance cost of medical treatment for residents of villages and townships was obtained, and the results have been visualized (Figure 2; Figure 3). Figure 2 shows that the distribution of the distance cost for medical treatment in rural areas first increased and then decreased, showing a significant normal distribution, and reaching a peak in the range of 4–6 km. The distance cost of medical treatment in most areas remains within 2–6 km. Specifically, 26.3% of the villages are in the range of 4–6 km, followed by 2–4 km (25.6%), below 2 km (12.0%), and the least in the range of 8–10 km (8.5%). The distance cost of medical treatment in most townships was in the range of 2–6 km, accounting for 81.4% of the total. Only 1.1% of the townships had a distance cost below 2 km, while 55 townships had a distance cost above 10 km, accounting for 3.4% of the total.

Figure 3 shows apparent spatial differences in medical accessibility in rural areas of Henan Province. Villages with good accessibility (distance cost < 1 km) are distributed throughout 18 prefectures and cities in Henan Province; however, the quantity differs. Among these, Xinyang has the largest number (1115), while Hebi has the lowest number (37). Villages with poor accessibility (distance cost > 20 km) are mainly concentrated in Nanyang, Luoyang, and Sanmenxia. On the whole, villages in the west have poor accessibility, while villages in the middle and east have better accessibility (Figure 3a).

At the township scale (Figure 3b), the distance cost of medical treatment gradually increased from east to west, indicating that regions with good accessibility were mainly distributed in the central and eastern regions, while the western regions had poor accessibility. Among these, the distance cost for medical treatment in Yingqiao Hui Town of Xiangcheng County was only 841.7 m, while that in Taiping Township of Xixia County was as high as 15807.9 m. The distance cost for medical treatment in most townships of Lingbao City, Jiyuan City, Mianchi County, Xin’an County, Lushi County, Songxian County, Xixia County, Nanzhao County, and Neixiang County in the western region exceeded 8988.8 m, which indicates poor accessibility for medical treatment for residents in these areas.

**Figure 4:**
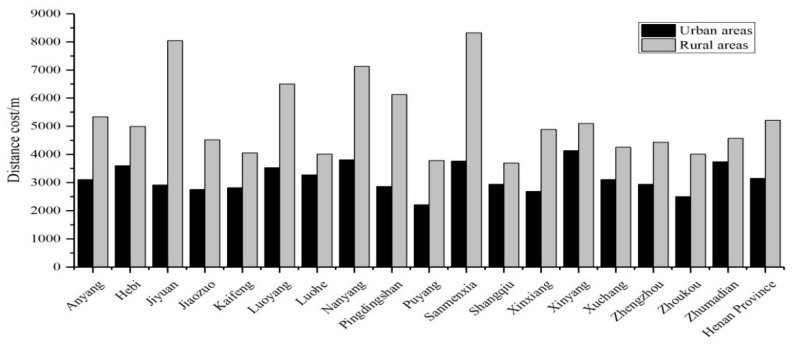
Differences in spatial accessibilities between urban and rural areas.

A big gap was found in spatial accessibility between urban and rural areas (Figure 4). On average, the distance cost in rural areas is 5211 m, while that in urban areas is 3148 m, indicating a difference of 2063 m. Among the 18 prefectures and cities in Henan Province, Jiyuan City has the largest gap between urban and rural areas, with a difference of 5134.7 m, followed by Sanmenxia City, with a difference of 4561.7 m. Luohe City has the smallest gap of 743.4 m. This gap between urban and rural areas is a reflection of the genuine problem for rural residents to seek medical treatment.

### 3.2. Spatial Equilibrium of Accessibility

**Figure 5:**
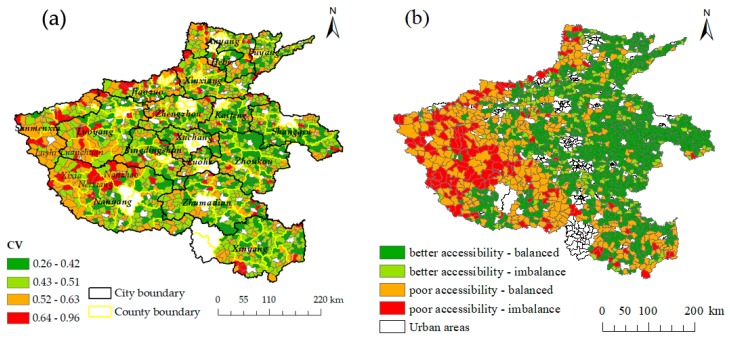
(**a**) Spatial pattern of the CV and (**b**) accessibility-equilibrium.

The CV reflects the equilibrium degree of accessibility of residential areas in a region. A higher CV indicates a worse equilibrium. Figure 5a shows that the equilibrium degree of accessibility of each township differs. The minimum CV is 0.26 and the maximum is 0.96. The CV of most townships ranged between 0.43 and 0.51, accounting for 43.0% of the total. 7.6% of the townships had a CV above 0.64. In general, the CV in the central and eastern regions are low, while the CV in the western region are generally larger, i.e., the equilibrium of the eastern and central regions is generally good, but the equilibrium of the western region is generally poor. The CV also reflects the spatial fairness of medical facilities in a region, and for regions with large CV, measures should be taken to improve the level of equalization of internal medical services.

The accessibility-equilibrium in rural areas of Henan Province is divided into four categories. As shown in Figure 5b, townships of the better accessibility-balanced type are the most abundant of the four types, accounting for 46.4% of the total, and are mainly distributed in the middle and eastern parts of Henan Province. The poor accessibility-imbalance type is lowest, accounting for 13.1% of the total, and these are mainly distributed in the northern and western mountainous areas. The better accessibility-imbalance type is mainly located in the central region and its distribution is spread. The distribution of poor accessibility-balanced type is more concentrated, and is mainly concentrated in the western part of Henan Province.

### 3.3. Spatial Autocorrelation Analysis

The global autocorrelation analysis of the spatial accessibility in townships indicated that the Moran’s I index was 0.5315, the Z value was 38.75, and the *p* value was 0.000, which passed the significance test. The results therefore indicated a significant spatial autocorrelation in medical accessibility in rural areas of Henan Province. Consequently, the spatial agglomeration characteristics of medical accessibility in township areas are significant.

**Figure 6:**
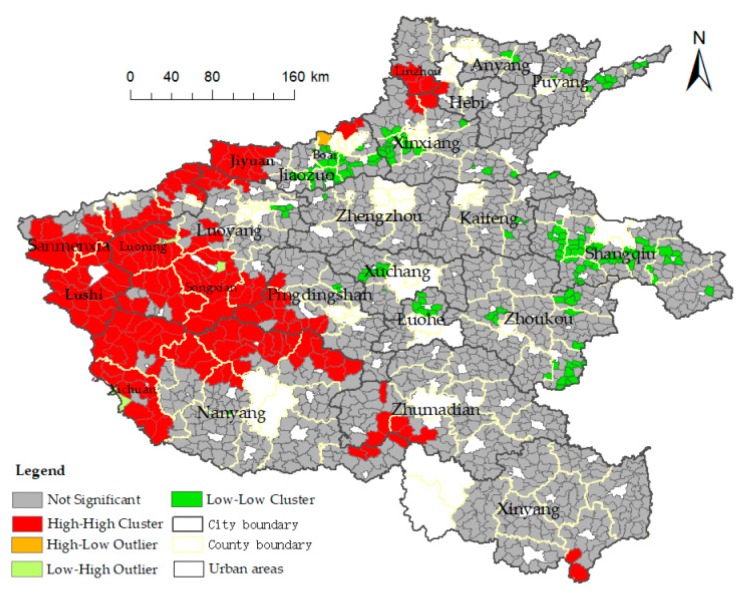
Local indicators of spatial association cluster map of spatial accessibility to medical facilities.

Moreover, local indicators of spatial association (LISA) were adopted to explain the similarity and correlation between the accessibility of each township and its adjacent space unit. As shown in Figure 6, the accessibility of the study area shows a clear phenomenon of high value area agglomeration and low value area agglomeration, and few areas have abnormal values. The H-H types are concentrated in Linzhou City in the north and Jiyuan, Sanmenxia, Luoyang, Pingdingshan, and parts of Nanyang in the west, and these areas are high-value synergy areas.

The L-L type is distributed in Xinxiang, Jiaozuo, Shangqiu, and Zhoukou, which were defined as low-value synergy areas. The L-H type is located in Luoning County, Songxian County, and Xichuan County in the west. The spatial accessibility of these areas is quite different from that of the surrounding areas. The H-L type is located in Boai County in the northwest, which is surrounded by low-value areas.

### 3.4. Spatial Heterogeneity Analysis Based on the GWR Model

To explore the reason underlying the observed inequality of spatial accessibility, this study analyzed the potential influencing factors with regard to the following three aspects: nature, society, and economy. Specifically, area and elevation were chosen as natural factors. The number of industrial enterprises, population density, road network density, and residential area density were used as social factors. The gross industrial output value and per capita industrial output were selected as a representation of the economic level.

#### 3.4.1. OLS Regression Analysis

Before using the GWR model, the explanatory variables were analyzed via OLS model regression analysis. Table 1 shows that the adjusted *R*^2^ in the regression results is 0.722, and the F-test is significant at the level of 5%, indicating a good fitting degree of the model. 

The VIF test values of the eight explanatory variables are all below 5; therefore, multicollinearity does not exist between the explanatory variables. The regression coefficient shows that area, per capita industrial output, and residential area density are positive indicators, while gross industrial output value, number of industrial enterprises, population density, and road network density are negative indicators. The P value indicates that the gross industrial output value and the number of industrial enterprises have not passed the significance test; therefore, they were eliminated in the regression analysis of the GWR model.

#### 3.4.2. Spatial Heterogeneity of the Influencing Factors

**Figure 7:**
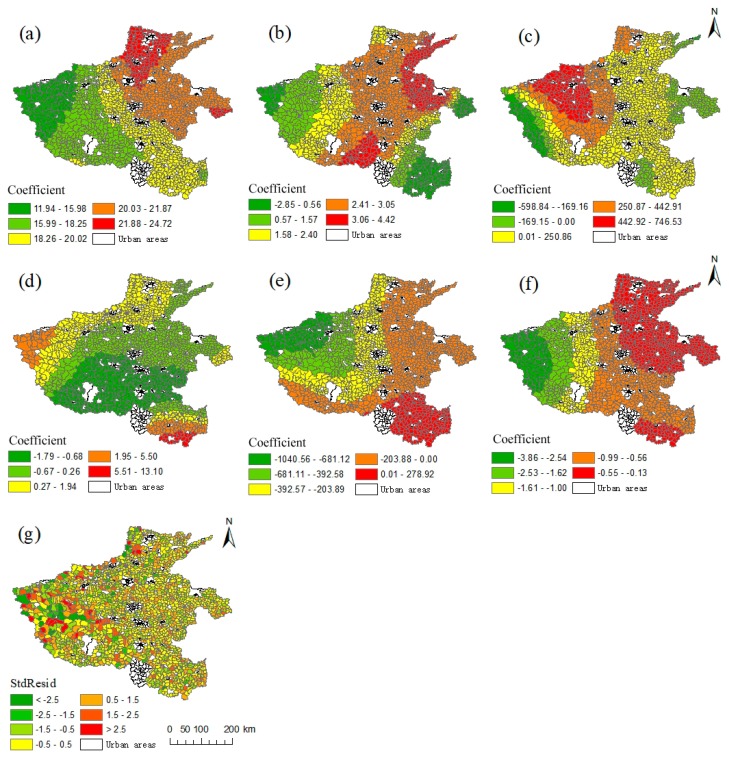
Spatial pattern of both regression coefficient and standardized residual of the GWR Model: (**a**) regression coefficient of the area; (**b**) regression coefficient of the elevation; (**c**) regression coefficient of the residential area density; (**d**) regression coefficient of the per capita industrial output value; (**e**) regression coefficient of the road network density; (**f**) regression coefficient of the population density; (**g**) standardized residuals.

The adjusted *R*^2^ in the regression results of the GWR model is 0.749, which exceeds that of the OLS model, indicating that the GWR model can better fit the observed data. In addition, compared to the OLS model, the AICc value decreased by 21.584, indicating that the GWR model is applicable. The pattern of spatial heterogeneity between spatial accessibility and its influencing factors is shown in Figure 7. The regression coefficients of the six explanatory variables have different distribution characteristics in space and the standardized residuals are completely randomly distributed.

Area has a significant impact on accessibility with an average regression coefficient of 19.47. In general, hospitals are concentrated in built-up areas of townships, while larger areas have more settlements that are far from township built-up areas. Therefore, the distance cost of medical accessibility is large for large areas. The spatial distribution of the regression coefficient increases gradually from west to east. The most affected area is located in the north of Henan Province. Here, the regression coefficient exceeds 21.88, while the degree of influence in the western region is small and only has a regression coefficient below 15.98.

Apparent spatial differences were found in the degree of influence of the elevation factor on spatial accessibility. The influence in the central region is stronger and gradually decreases from the center toward the east-west direction (Figure 7b). The most affected areas are distributed in the continuous zone of Puyang-Kaifeng-Shangqiu, as well as in Tongbai County in Nanyang and in Biyang County in Zhumadian. The regression coefficients of these areas exceeded 3.06. Less affected areas are located in Sanmenxia and Luoyang in the west and in Xinyang in the southeast, with regression coefficients below 1.57.

The average regression coefficient of the residential area density was 149.5 and showed a significant positive correlation. The residential area density is high, i.e., the number of settlements per unit area is large; consequently, this will aggravate the competition for medical resources, and thus, reduce their level of accessibility. The regression coefficient of residential area density has a significant spatial difference, and the most affected areas are Luoyang, Jiaozuo, Sanmenxia, and Pingdingshan. Their regression coefficients all exceeded 442.92. Road network density has a significant negative correlation effect with an average regression coefficient of −221.6. The high road network density indicates the perfection of road facilities, which significantly impacts the spatial accessibility. The regression coefficient decreases gradually from east to west, and the western region has the highest negative correlation effect with a regression coefficient below −392.58 (Figure 7e).

The population density showed a significant negative correlation, and the average regression coefficient was −0.86. This is because the densely populated areas typically have more medical facilities, resulting in more convenient access of medical services. The spatial distribution of the regression coefficient of population density gradually decreases from east to west, i.e., the negative correlation degree of population density is strongest in the western region. Compared to other indicators, the impact of the per capita industrial output on medical accessibility is relatively small, with an average regression coefficient of 0.08. The per capita industrial output value exerts a relatively strong negative impact on Nanyang, Pingdingshan, Luohe, and Zhumadian, and a relatively strong positive influence on the south of Xinyang City.

## 4. Discussion

Inequality and imbalance of accessibility of medical facilities were found across 203801 villages and 1609 townships in Henan Province. The distance cost of medical treatment was less than 6 km for most villages, while the distance cost in more than 10% of all villages exceeded 10 km. These 10% of the villages all have a common feature, i.e., they are far from built-up areas of townships. Therefore, to reduce the spatial difference of the accessibility of medical facilities, more health service facilities should be built and distributed in villages far from built-up areas of townships [5,41]. The accessibility of medical facilities in the eastern and central regions of Henan Province is good, while that in the western region is poor. The main reasons for this spatial difference are the prevailing terrain and elevation [30,33,42]. The central and eastern parts of Henan Province are mostly plain areas and feature better medical accessibility. However, from the northern Taihang Mountains to the Funiu Mountain-Xionger Mountain area in the west and part of Tongbai Mountain-Dabie Mountain areas in the south, the distance cost of medical treatment is relatively high. Consequently, the level of accessibility is poor. This is due to the undulating mountainous terrain, with its rugged roads and inadequate road network, both of which affect accessibility. For example, Songxian County and Lingbao City have poor accessibility (Figure 3b), and their mountainous and hilly areas account for more than 90% of the total area. The CV of Jiyuan City is large, which is mainly due to the large average altitude gap between the North and the South (Figure 5a), which causes an imbalance of spatial accessibility.

Policy assessments should not only estimate geographic accessibility but should also aim to increase unbiased resource distribution [27]. China’s rules for the allocation of public services emphasize equality between urban and rural areas, as well as equality between administrative regions at the same level [43]. This study introduced the CV to analyze the equality of residents’ access to health care. The results showed that the CV of the central and eastern regions of Henan Province are small, while the CV of the western region is large. This is related to the large area occupied by townships and the scattered distribution of settlements in the western region [6,22]. The CV values of Luanchuan County, Neixiang County, Xixia County, Lushi County, and Nanzhao County are even larger (Figure 5a). These areas have fewer roads, which greatly affects the accessibility of medical facilities and thus increases inequality [42]. According to the results of accessibility and CV, the accessibility and equilibrium of rural areas was divided into four categories. This provides a solution for the difficulty to assess the lack of medical treatment and unbalanced areas, as well as aiding the rational layout and optimization of medical resources.

The accessibility to medical facilities in rural areas of Henan Province shows a clear spatial autocorrelation. The HH-type, cluster of poor spatial accessibility to medical facilities, matches the complex topography and underdeveloped economic conditions well. Representative areas are Lushi County, Luoning County, Song County, and Xichuan County. These areas are located in mountainous areas with high average altitude and relatively underdeveloped economic level. It should be noted that the L-H type areas are also located in Luoning County, Xichuan County, and Song County. These areas are closer to the county seat, with more medical resources and more convenient transportation facilities. Thus, these form a phenomenon where low-value areas are surrounded by high-value areas. The LL-type, cluster of good spatial accessibility to medical facilities, are mainly distributed throughout Shangqiu, Xinxiang, Jiaozuo, Xuchang, and Luohe. Most of these areas are located on flat terrain with low average elevation. Shangqiu City is the transportation hub of Henan Province, with perfect road facilities and good road accessibility. Xinxiang, Jiaozuo, Xuchang, and Luohe have better economic development and better investment in public health. The H-L type areas are located in Boai County, Jiaozuo City. Boai County is located in the Taihang Mountain area, which features a complex topography. The surrounding area is relatively flat, thus forming a phenomenon of high-value areas surrounded by low-value areas.

Few current studies have addressed the factors that affect the accessibility of medical facilities, especially in rural areas. The present study explored the influencing factors of accessibility in rural areas from three aspects: nature, society, and economy. The results show that area, elevation, residential area density, per capita industrial output, road network density, and population density significantly impact the accessibility of rural areas. Thus, the government (or other relevant departments) should consider influences of local conditions, and propose improvement measures accordingly when they begin to process the spatial allocation of medical facilities or the optimization of their spatial layout. The conducted GWR analysis explored the extent of the impact and the direction of different types of influencing factors at the administrative level. Significant spatial variability was found among most levels. The results of GWR regression indicate that the residential area density greatly influences the accessibility, especially in Luoyang, Jiaozuo, Sanmenxia, and Pingdingshan. Therefore, the rational planning of rural settlements and the promotion of moderate concentration of rural population are conducive to the improvement of the medical accessibility of rural areas. The road network density exerts the strongest impact on the western region. Therefore, with the existing medical resources, the western region should focus more on improving the road infrastructure to improve regional medical accessibility [42,44]. In addition, the per capita industrial output exerts less impact on accessibility, indicating that the direct impact of the economy is weak, and more attention should be focused on infrastructure construction and the optimal distribution of medical resources. Although the GWR model may suffer from over-fitting, it indicates that the relationship between accessibility and influencing factors is spatial heterogeneity and non-stationarity, which aids the government (or relevant departments) to formulate improvement measures according to local conditions.

Henan serves as a microcosm of the broader state of China. Problems and solutions associated with access to and inequity in medical services in Henan are also likely applicable across the other provinces of China. At present, most of the studies on accessibility use streets or townships as basic research units, while this study uses unincorporated villages as basic research unit. Therefore, the results have more practical significance. This study investigated the differences of accessibility to medical services in rural areas using the convenience of residents’ access to these. The scale and hierarchy of medical facilities has not been considered, and further studies are required to investigate the differences in the accessibility of medical facilities at different scales.

## 5. Conclusions

Based on GIS network analysis and using unincorporated villages as basic unit, this study explored the spatial characteristics of accessibility to medical facilities and its equilibrium in rural areas of Henan Province, China. Both the CV method and the spatial analysis method were used, and the spatial heterogeneity of influencing factors is discussed by the using GWR model. The major findings of this study can be summarized as follows: Obvious spatial differences exist in the accessibility of medical facilities in rural areas. In general, the eastern and central regions have better spatial accessibilities, while the western region has poor spatial accessibility. In particular, the medical accessibility of mountainous areas had clear disadvantages. The difference of the equilibrium degree at the township scale was also obvious. The equilibrium degree in the western region is relatively poor. Moreover, a significant spatial agglomeration characteristic was found in the study area and several prominent counties were detected. Furthermore, the effects of area, elevation, residential area density, per capita industrial output, road network density and population density on the accessibility showed clear spatial heterogeneity.

The results indicate the characteristics of the spatial distribution and pattern of the medical treatment imbalance in rural areas in China, thus enriching the available knowledge on the equity of the infrastructure and providing a reference for policy making that will aid the improvement of the unbalanced development. Therefore, this study suggests that the government should focus on the phenomenon of the unequal accessibility of medical facilities in rural areas and actively promote a regionally balanced development. This is particularly important for the balanced development of infrastructure related to people’s life. Moreover, policy makers should take effective measures and integrate local conditions, thus enabling a reduction of in-intra region and inter-region differences. Further studies should combine the spatial accessibility model with the planning of medical facilities, solve the optimization of the layout of rural medical resources, and provide suggestion for reducing the unfairness of rural medical services.

## Figures and Tables

**Table 1:** Parameter estimation and test results of the OLS model.

Factors	Coefficient	*p*	VIF
Area	17.982	0.000 *	2.199
Gross industrial output value	−0.000134	0.249	4.042
Number of industrial enterprises	−0.106436	0.309	1.194
Elevation	2.643	0.000 *	1.897
Per capita industrial output	0.063	0.034 *	3.762
Population density	−0.909	0.000 *	2.129
Road network density	−188.137	0.002 *	1.481
Residential area density	118.689	0.015 *	1.076
Adjusted *R*^2^	0.722		
*F*-statistics	522.366		
*F*-test	0.000 *		
AICc	26927.965		

* It is significant at a level of 10%.
